# Biotin-Containing Third Generation Glucoheptoamidated Polyamidoamine Dendrimer for 5-Aminolevulinic Acid Delivery System

**DOI:** 10.3390/ijms22041982

**Published:** 2021-02-17

**Authors:** Aleksandra Kaczorowska, Małgorzata Malinga-Drozd, Wojciech Kałas, Marta Kopaczyńska, Stanisław Wołowiec, Katarzyna Borowska

**Affiliations:** 1 Department of Biomedical Engineering, Faculty of Fundamental Problems of Technology, Wrocław University of Science and Technology, 27 Wybrzeże Wyspiańskiego Str., 50-370 Wrocław, Poland; aleksandra.kaczorowska@pwr.edu.pl (A.K.); marta.kopaczynska@pwr.edu.pl (M.K.); 2 Medical College, University of Rzeszów, Warzywna 1a, 35-310 Rzeszów, Poland; malgorzatamalinga@wp.pl; 3 Department of Experimental Oncology, Ludwik Hirszfeld Institute of Immunology and Experimental Therapy, Polish Academy of Sciences, Rudolfa Weigla 12 Str., 53-114 Wrocław, Poland; wojciech.kalas@hirszfeld.pl; 4 Department of Histology and Embryology with Experimental Cytology Unit, Medical University of Lublin, 11 Radziwiłowska Str., 20–080 Lublin, Poland; k_borowska@wp.pl

**Keywords:** photosensitizer delivery system, PAMAM dendrimer, photodynamic therapy, cytotoxicity, phototoxicity, colorectal adenocarcinoma

## Abstract

Polyamidoamine PAMAM dendrimer generation 3 (G3) was modified by attachment of biotin via amide bond and glucoheptoamidated by addition of α-D-glucoheptono-1,4-lacton to obtain a series of conjugates with a variable number of biotin residues. The composition of conjugates was determined by detailed 1-D and 2-D NMR spectroscopy to reveal the number of biotin residues, which were 1, 2, 4, 6, or 8, while the number of glucoheptoamide residues substituted most of the remaining primary amine groups of PAMAM G3. The conjugates were then used as host molecules to encapsulate the 5-aminolevulinic acid. The solubility of 5-aminolevulinic acid increased twice in the presence of the 5-mM guest in water. The interaction between host and guest was accompanied by deprotonation of the carboxylic group of 5-aminolevulinic acid and proton transfer into internal ternary nitrogen atoms of the guest as evidenced by a characteristic chemical shift of resonances in the ^1^H NMR spectrum of associates. The guest molecules were most likely encapsulated inside inner shell voids of the host. The number of guest molecules depended on the number of biotin residues of the host, which was 15 for non-biotin-containing glucoheptoamidated G3 down to 6 for glucoheptoamidated G3 with 8 biotin residues on the host surface. The encapsulates were not cytotoxic against Caco-2 cells up to 200-µM concentration in the dark. All encapsulates were able to deliver 5-aminolevulinic acid to cells but aqueous encapsulates were more active in this regard. Simultaneously, the reactive oxygen species were detected by staining with H2DCFDA in Caco-2 cells incubated with encapsulates. The amount of PpIX was sufficient for induction of reactive oxygen species upon 30-s illumination with a 655-nm laser beam.

## 1. Introduction

Photodynamic therapy (PDT) is a non-invasive and an effective procedure that has been clinically approved for treating a number of diseases, including cancer. PDT is widely used in dermatology in the treatment of actinic keratoses [1], Bowen’s disease [2,3], and cutaneous microbial infections, for example, acne, onychomycosis, and verrucae. [4]. With its range of indications continually expanding, PDT has also demonstrated potential as a treatment for dermatological malignancies such as squamous cell carcinoma [5] and superficial basal cell carcinomas [6]. In addition, PDT has been applied in treatment with other types of human cancers such as cervical cancer [7], breast cancer [8], glioma [9], prostate cancer [10], and colorectal cancer (CRC) [11,12]. CRC is one of the most common diagnosed cancers and one of the leading causes of death worldwide [13]. Due to great metastatic potential and high invasiveness, both radical and selective methods for CRC treatment are required [14]. The method that meets these criteria and can be used in personalized therapy of CRC is PDT [15]. One of the most common agents used in PDT of colorectal with satisfactory results cancer is 5-aminolevulinic acid (ALA), a precursor of Protoporphyrin IX (PpIX) [14,16].

PDT offers the advantages of minimal invasiveness, better cosmetic outcomes, and minimal functional disturbances. PDT is usually well tolerated and can be applied repeatedly at the same site [17]. The efficacy of PDT depends on the level of reactive oxygen species (ROS), such as singlet oxygen generated by photosensitizers (for example ALA) upon specific laser irradiation to induce tumor cell apoptosis and/or necrosis [18,19]. ALA has been shown to be effective at inducing PpIX after topical, oral, and intravenous applications in vivo [20]. Although ALA is widely used in PDT, cellular uptake of ALA is limited by its solubility and ability to penetrate biological barriers [21]. The efficiency of PDT is then far from satisfactory as optimal tissue accumulation and localization of ALA remains a clinical problem. Although it was shown that ABCG2 is an important efflux transporter of PpIX, especially in glioma cells [22] limiting intracellular PpIX concentration, the mechanism of PpIX accumulation is far more complex and involves the ALA influx and biosynthesis rate [23]. But in the case of Caco-2, the role of ABCG2 in transport of its other known substrates in cells is unclear [24], underlining a wide spectrum of influx/efflux mechanisms. 

In order to avoid protein-assisted ALA influx barrier, we used dendrimeric drug carriers which showed an appreciable efficacy for ALA delivery [25,26,27]. Thus, we modified polyamidoamine (PAMAM) G3 and synthesized encapsulates with ALA. The cationic character of host PAMAM was eradicated by primary amine groups substitution with glucoheptoamide substituents. In addition, biotin was covalently attached into amine groups in order to facilitate selective binding of dendrimer into cell membrane of Caco-2, which is one of many cancer cells with overexpression of biotin receptors [28]. It has been previously shown that biotin-attached glucoheptoamidated PAMAM G3 dendrimer accumulated four times more effectively in fibroblasts (BJ), squamous cell carcinoma (SCC-15), and glioblastoma (U-118MG) cells than non-biotinylated analogues, in a time- and concentration-dependent manner [29]. Biotin-attached dendrimers were also less toxic than non-biotinylated analogues within 10–50 µM concentrations for all cell lines. We adopted these molecules as the host of ALA guest and tested the encapsulates for photocytotoxicity. 

## 2. Results and Discussion

### 2.1. Chemistry

#### 2.1.1. Synthesis and Characterization of Modified PAMAM G3 Dendrimers

**Figure 1:**
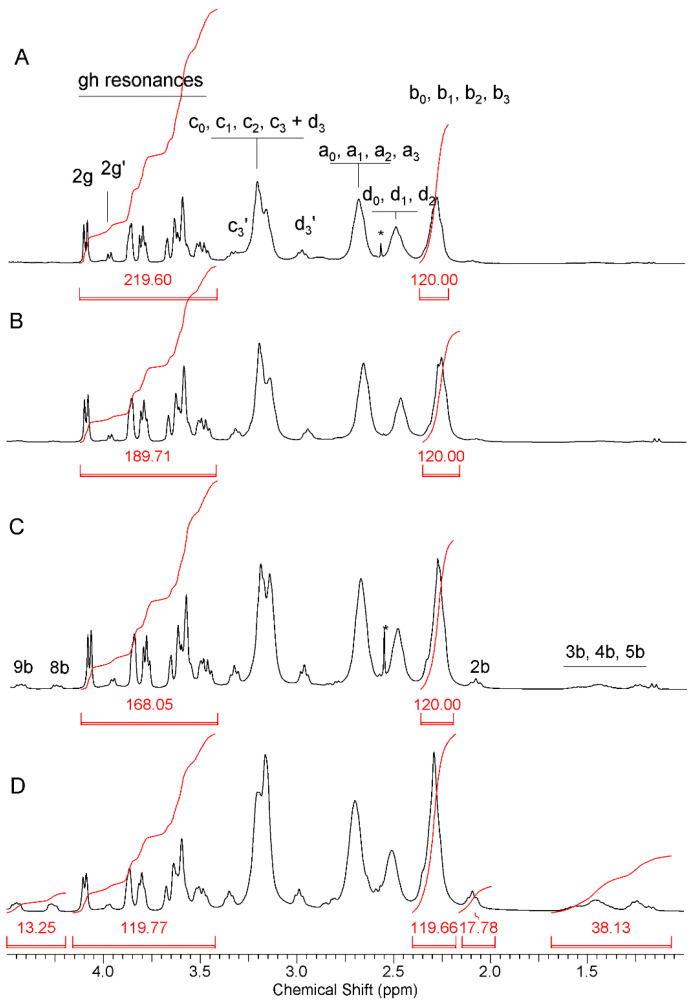
The ^1^H NMR spectra of G3^1B31gh^ (**A**), G3^2B27gh^ (**B**), G3^4B24gh^ (**C**), and G3^8B17gh^ (**D**) in deuterium oxide. The broad signal for all CH_2_(b) resonances of the PAMAM G3 core was used as internal intensity reference [120H]. The residual DMSO resonance is labeled with asterisk.

PAMAM G3 dendrimer was modified by addition of α-*D*-glucoheptono-1,4-lactone (GHL) and biotin (Figure 1). Both substituents were amide-bonded to terminal (surface) primary amine groups of G3 in order to eradicate the cationic character of PAMAM G3 in neutral aqueous solution. 

**Scheme 1:**
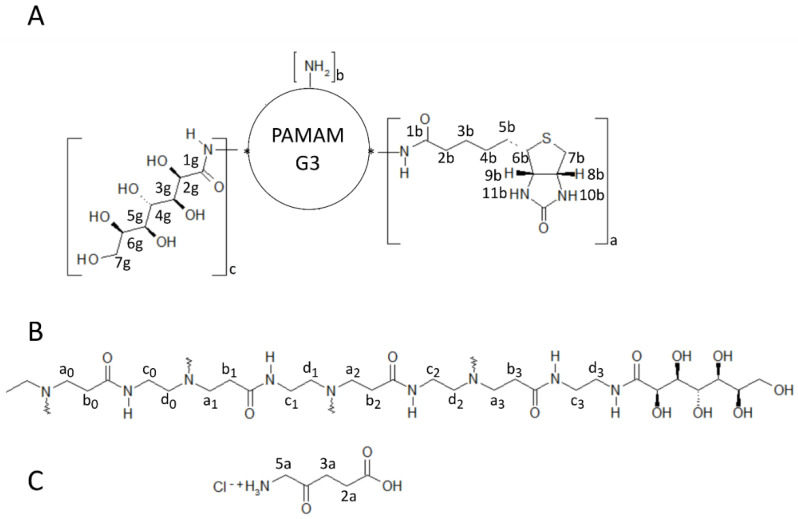
General formula of polyamidoamine (PAMAM) generation three (G3) conjugates used herein with atom numbering of glucoheptoamide and biotin substituents (**A**) and one arm of PAMAM G3 starting from nitrogen atom of ethylenediamine core (**B**). The following conjugates were synthesized: G3^1B31gh^ (a = 1, b = 0, c = 31); G3^2B27gh^ (a = 2, b = 3, c = 27); G3^4B24gh^ (a = 4, b = 4, c = 24); G3^6B21gh^ (a = 6, b = 5, c = 21); G3^8B17gh^ (a = 8, b = 7, c = 17). Atom numbering for 5-aminolevulinic acid (in protonated form) is given in (**C**).

**Figure A1:**
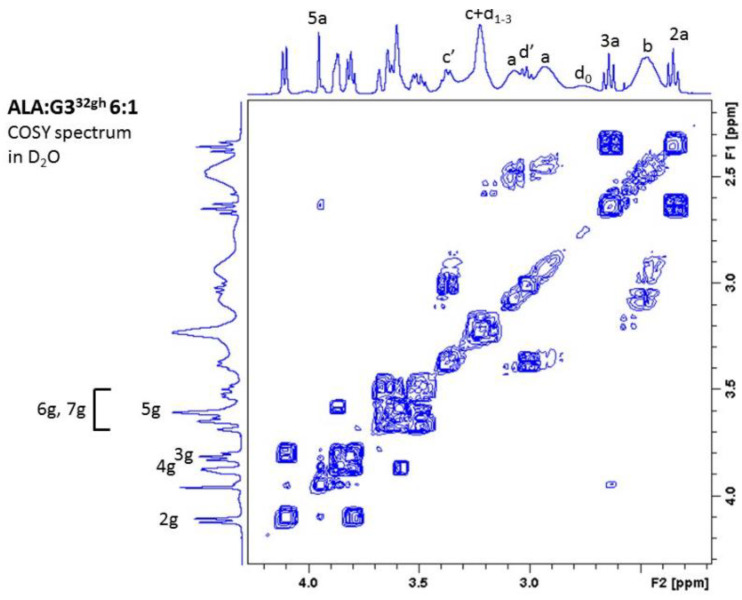
^1^H-^1^H- COSY spectrum of G3^32gh^ in D_2_O.

**Figure A2:**
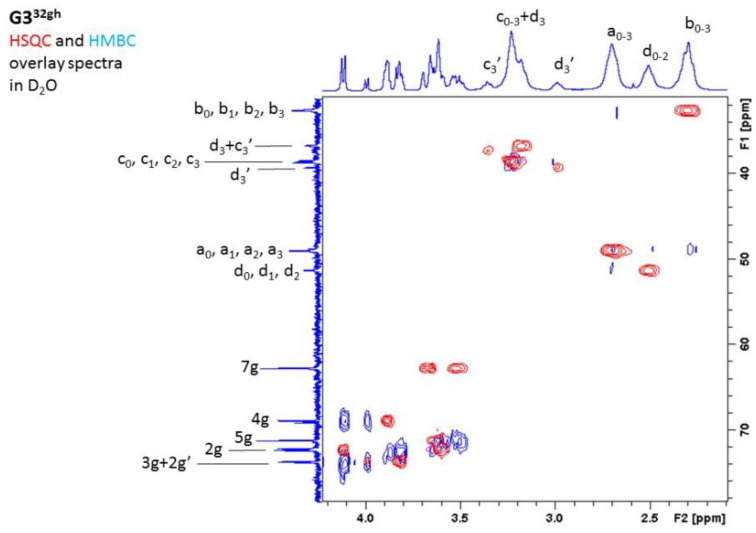
^1^H-^13^C HSQC (red cross-peaks) and HMBC (blue cross-peaks) spectra of G3^32gh^ in D_2_O.

The conjugates were characterized by NMR spectroscopy. The ^1^H NMR spectra of conjugates allowed us to determine the average stoichiometry of the conjugates by integration of PAMAM resonances which covered the 2.2–3.4 ppm region, versus glucoheptoamide (gh) –CH resonances which were multiplets within the 3.4–4.2 ppm region, and versus biotin resonances, some of which were well behind the G3 and gh resonances envelope. The multiplets of 3b, 4b, and 5b (total of six protons) were in the region 1.0–1.7 ppm and 8b and 9b quartets at 4.34 and 4.25 ppm, respectively (Figure 1; for atom numbering see also Scheme 1). Considering that all PAMAM CH_2_(b) signals (triplets) were overlapped and located at 2.3 ppm, this resonance of intensity [120H] was used as an internal intensity reference. Other PAMAM G3 resonances were assigned as before [29]. Two sets of –CH resonances were observed for gh substituents, which were combined by scalar coupling in 2-D ^1^H-^1^H correlations spectroscopy (COSY), ^1^H-^13^C heteronuclear single quantum correlation (HSQC), and heteronuclear multiple bond correlation (HMBC) experiments (Figure A1 and Figure A2 (Appendix A), respectively). Two doublets of 2g protons were observed at 4.10 and 3.96 ppm with a 3.63:1 intensity ratio. Such a considerable chemical shift difference only at 2g resonance suggested that gh substituents are bound by trans- and cis-amide bonds into terminal amine groups of G3. Considering the intensity of 2g and 2g’ doublets the average 26 gh substituents were trans- and 6 gh substituents are cis-amide-bonded in G3^32gh^. Moreover, two triplet resonances from the G3 terminal ethylene group, namely d_3_’ and c_3_’ (Figure 1) had a double intensity of the 2g’ resonance. The patterns of major and minor resonances from gh occurred reproducibly in all compounds obtained here, i.e., biotin-containing conjugates G3^1B31gh^, G3^2B27gh^, G3^4B24gh^, G3^6B21gh^, and G3^8B17gh^, as well as in various generation glucoheptoamidated PAMAM glycodendrimers, G2^16gh^, G4^64gh^, and G5^128gh^.

The average stoichiometry of G3^1B31gh^, G3^2B27gh^, G3^4B24gh^, G3^6B21gh^, and G3^8B17gh^ was determined by integration of gh –CH resonances versus internal reference of the PAMAM G3 CH_2_(b) signal at 2.30 ppm corresponding to [120H]. Thus, in the series of G3^1B31gh^, G3^2B27gh^, G3^4B24gh^, G3^6B21gh^, and G3^8B17gh^, the intensities gh –CH resonances were: 220, 190, 168, 147, and 120, which after dividing the intensity by 7 (number of –CH protons in every gh substituent) gave 31, 27, 24, 21, and 17 gh substituents per conjugate molecule, respectively (Figure 1).

#### 2.1.2. Interaction of 5-Aminolevulinic Acid with G3^Bgh^ Conjugates; Stability of ALA@G3^gh^ Encapsulates

**Figure 2:**
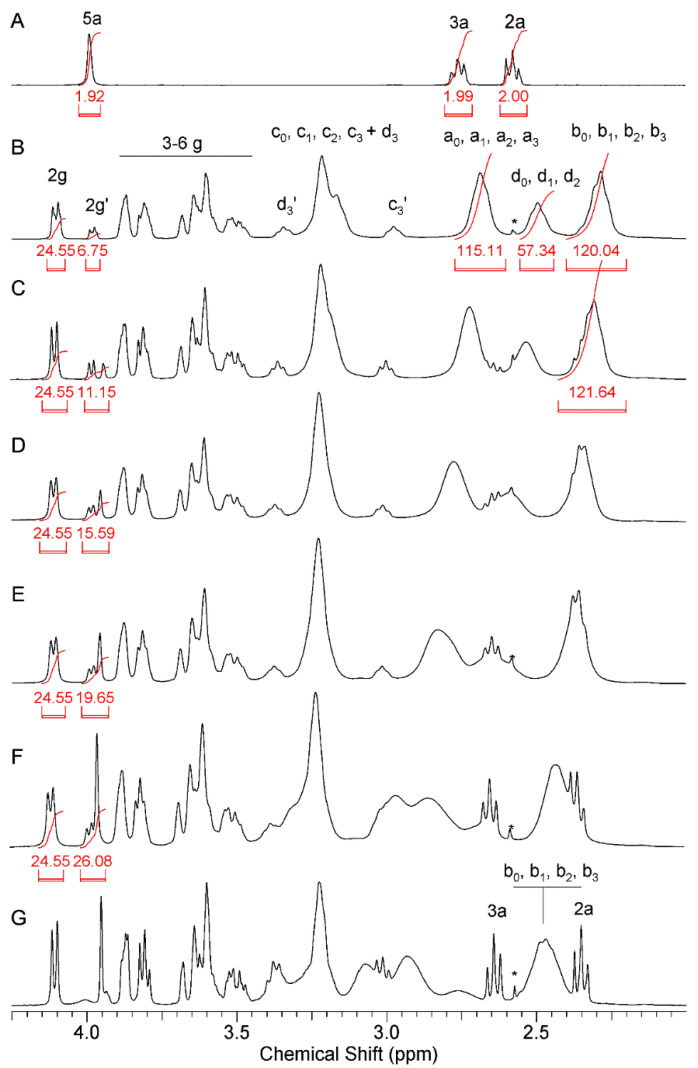
The ^1^H NMR spectra of (**A**) 5-aminolevulinic acid (ALA) in D_2_O; (**B**) G3^32gh^ (5.1 mM in D_2_O); (**C**–**F**) G3^32gh^ (5.1 mM in D_2_O) containing 2.2 (**C**), 4.4 (**D**), 6.5 (**E**), 9.7 (**F**), and 15 (**G**) equivalents of ALA. Final concentration of ALA in case of solution F was 76 mM, which is twice compared to ALA concentration in saturated aqueous solution. The residual DMSO resonance is labeled with asterisk.

**Figure A3:**
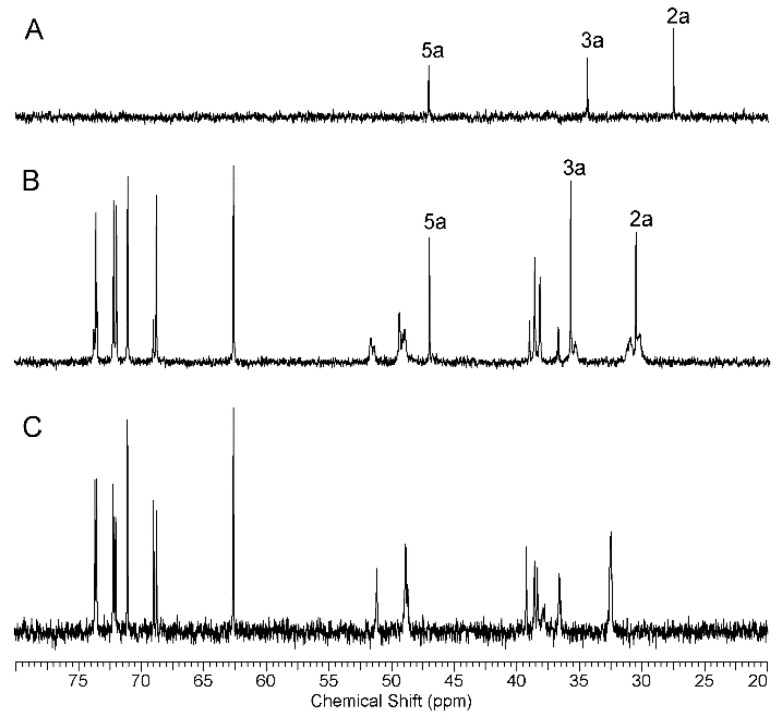
Aliphatic region of ^13^C NMR spectra of ALA (**A**), G3^32gh^ an d 8 equivalents of ALA (**B**), and G3^32gh^ (**C**) in D_2_O.

**Figure A4:**
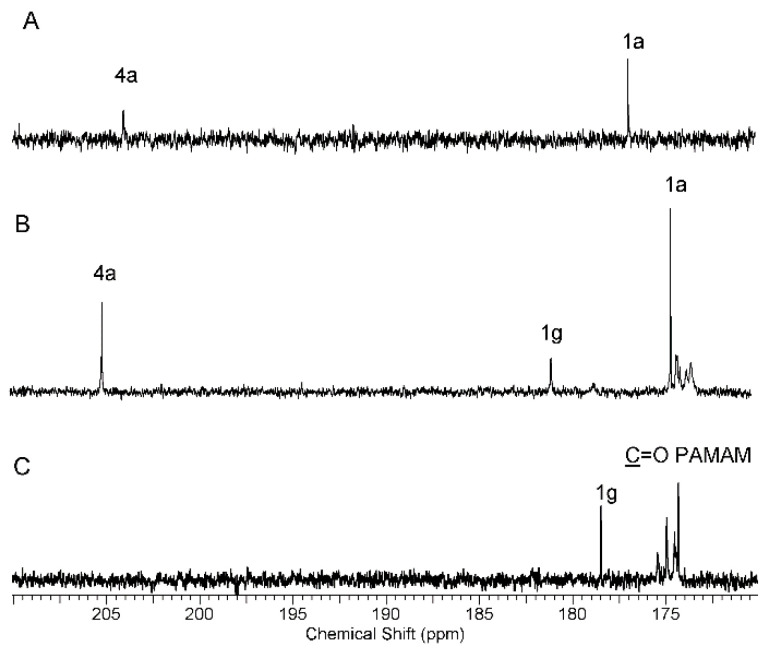
Downfield region of ^13^C NMR spectra of ALA (**A**), G3^32gh^ and 8 equivalents of ALA (**B**), and G3^32gh^ (**C**) in D_2_O.

ALA in aqueous solution occurs in the zwitterion form in pH between 5 and 7, according to determined pK_a_^(COOH)^ = 3.90 and pK_a_^(NH3)^ = 8.05 [30]. When this compound was added into the 5.1 mM solution of G3^32gh^, all the ^1^H and ^13^C resonances in NMR spectra of ALA shifted remarkably and remained unchanged until 68 mM concentration. The ^1^H NMR spectra of the solutions are presented in Figure 2 (titration experiment). Thus, the singlet CH_2_(5), and CH_2_(3) and CH_2_(2) resonances (5a, 3a, and 2a in Figure 2A) centered at 4.00, 2.77, and 2.58 for ALAxHCl shifted upfield to 3.96, 2.65, and 2.36 ppm, respectively, in the presence of G3^32gh^ (Figure 2B–G). The largest was shift δ2a = −0.22 ppm compared to Δ3a = −0.12 ppm and negligible Δ5a = −0.04 ppm. This suggests the involvement of carboxylic group of ALA in interaction with G3^32gh^ host molecule. Similar chemical shift od ^13^C resonances were observed, with ΔC2 = −2.7 ppm, ΔC3 = −1.4 ppm, ΔC5 = 0.0 ppm, ΔC4 = 1.0 ppm, and ΔC1 = 2.4 ppm (Figure A3 and Figure A4).

The largest chemical shifts for carbon and proton nuclei next to carboxylic group in the ^1^H and ^13^C NMR spectra accompanying the interaction of ALA with G3^32gh^ indicated clearly that the carboxylic group underwent deprotonation.

Moreover, the ^1^H NMR spectra of the G3 core changed regioselectively upon interaction with ALA with G3^32gh^. Namely, the –CH_2_- proton resonances neighboring ternary nitrogen atoms shifted downfield gradually upon addition of ALA. The common resonance of d_0_, d_1_, and d_2_ as well as a_0_, a_1_, a_2_, and a_3_ (all methylene groups of internal shells) shifted from 2.49 and 2.65 (Figure 2B) into eventually 2.93 and 3.07 ppm (Figure 2G), respectively. In addition, the resonances of b protons of the PAMAM G3 core shifted downfield from 2.27 into eventually 2.46 ppm, while resonances of c protons and all gh CH protons remained unaltered after the addition of ALA into solution containing G3^32gh^ in deuterium oxide.

**Figure 3:**
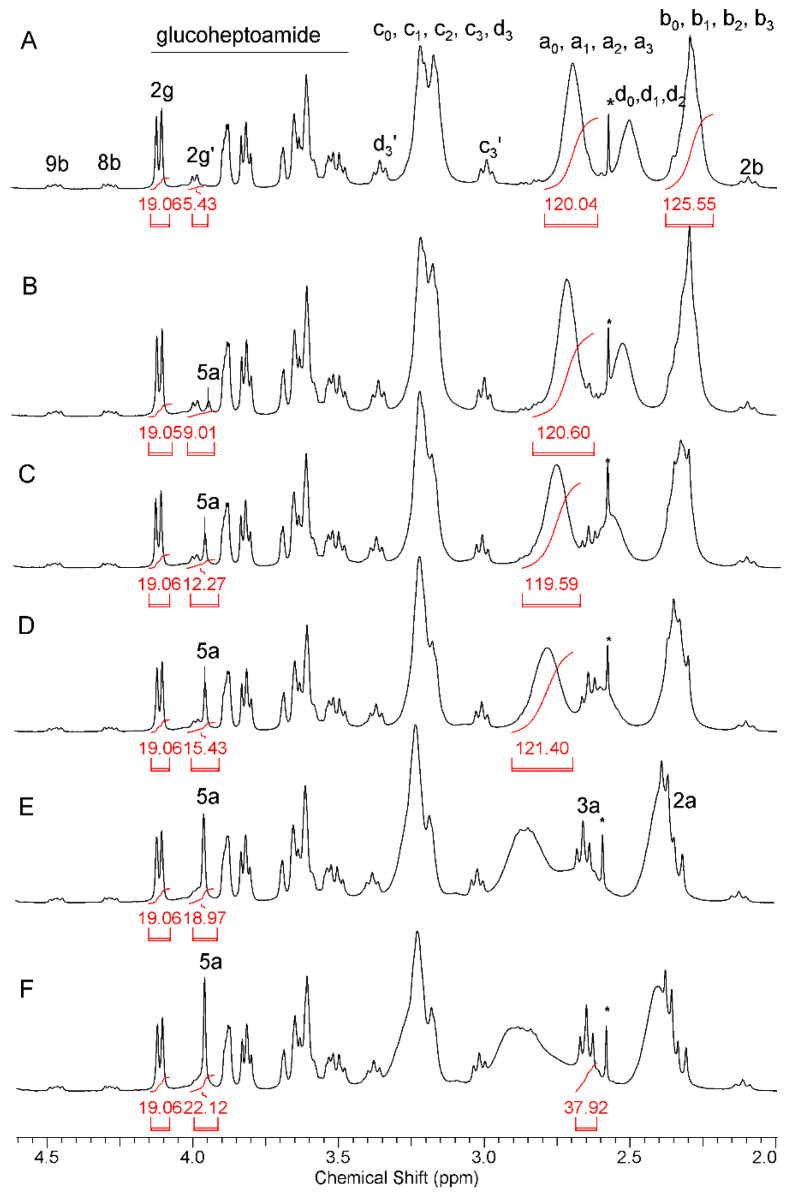
The ^1^H NMR spectra of (**A**) G3^4B24gh^ in D_2_O; (**B**–**F**) G3^4B24gh^ (6.2 mM in D_2_O) containing 1.8 (**B**), 3.4 (**C**), 5.0 (**D**), 8.0 (**E**), and 10 (**F**) equivalents of ALA. Final concentration of ALA in case of solution E was 62 mM, which is 1.7 higher if compared to ALA concentration in saturated aqueous solution. The residual DMSO resonance is labeled with asterisk.

Similar NMR spectral patterns of solutions containing G3^1B31gh^, G3^2B27gh^, G3^4B24gh^, G3^6B21gh^, and G3^8B17gh^ conjugates and ALA were observed. The ^1^H NMR spectra of the G3^4B24gh^ solution titrated with ALA are presented in Figure 3. The downfield shifts of PAMAM G3 core methylene proton resonances a, d, and b were consistent with protonation of ternary nitrogen atoms involved in dendrimer branching (see Scheme 1), while gh and B resonances remained unaltered upon addition of ALA. In addition, the methylene resonances d_3_’ and c_3_’ (outer sphere of G3 with cis-glucoheptoamidated amine groups) did not shift upon addition of ALA (Figure 3). This is presumably because the neighboring ternary nitrogen atoms are not involved in protonation due to steric hindrance of cis-gh groups which can be folded inside the outer sphere dendrimer cavity. In fact, four kinds of cavities in synthesized G3 conjugates could be expected based upon the formula of G3 conjugates, i.e., the number of them corresponding to branching nitrogen atoms: 2 in zero sphere, 4 in first sphere, 8 in second sphere, and finally 16 in third (outer-)sphere, some of the latter occupied by cis-gh substituents, which reduces the number of outer-sphere voids into 12. In total, the number of voids would then be 28, while the number of ternary nitrogen atoms, which are proton acceptors is 30. Thus, G3^32gh^ could accept 30 proton cations from 15 HClxALA donors and encapsulate 15 anionic ALA carboxylates. In fact, we found such final stoichiometry for the ALA:G3^32gh^ solution. In the case of the G3^1B31gh^, G3^2B27gh^, G3^4B24gh^, G3^6B21gh^, and G3^8B17gh^ conjugates, the voids’ availability was lower due to increasing steric hindrance imposed by B substituents, which not only enable total substitution of the remaining primary amine groups of G^B^ conjugates with GHL, but also reduce the ability of a conjugate to encapsulate ALA. Thus, the overall capacity of ALA encapsulation estimated by NMR spectral monitoring was 12, 10, 8, 7, and 6 equivalents of ALA per one equivalent of G3^1B31gh^, G3^2B27gh^, G3^4B24gh^, G3^6B21gh^, and G3^8B17gh^ conjugates, respectively. The numbers corresponded to 5-mM solutions of conjugates in water and can be even higher, although the encapsulates became water insoluble in the case of 7-mM solutions of the conjugates. Nonetheless, the encapsulates containing 6 equivalents of ALA were soluble in dimethylsulfoxide and water, and stable as it was evidenced by exhaustive dialysis with water. Therefore, we prepared the solutions of all conjugates in dimethylsulfoxide at a 6-mM dendrimeric host and 36-mM ALA guest and initially used them as stock solutions for biological tests (6ALA@G3^Bgh^). However, the stock solutions in DMSO were finally replaced by aqueous stock solutions (2.5 mM host G3^2B27gh^ or G3^6B21gh^, containing 10 mM ALA; 5ALA@G3^Bgh^ encapsulates) for phototoxicity studies in order to avoid interference from water–DMSO mixtures on metabolic behavior of the colorectal adenocarcinoma model line, i.e., Caco-2 cells. For simplicity the encapsulates would be further abbreviated as A@D^n^ (where n—the number of biotin moieties in the conjugate, D—glucoheptoamidated PAMAM G3 dendrimer).

The encapsulation of ALA by conjugates was accompanied by an increase of molecular size of conjugates, comparable to those observed for protonation. Thus, the number-average size determined by dynamic light scattering (DLS) of G3^32gh^ in water was 1.8 (±0.2) nm, while the molecule expanded into 4.0 (±0.2) nm at pH 5 [31]. The molecules of G3^32gh^, G3^2B27gh^, and G3^6B21gh^ expanded from 2.0 (±0.2) nm in water into 5.0, 5.3, and 5.4 (±0.3) nm upon addition of 5 equivalents of ALAxHCl and further for solution containing 16 equivalents of ALA. This was attributed to both protonation of tertiary amine groups and encapsulation of ALA.

### 2.2. Biological Studies 

#### 2.2.1. Cytotoxicity of Glucoheptoamidated PAMAM-Biotin Conjugates and ALA Encapsulates

**Figure 4:**
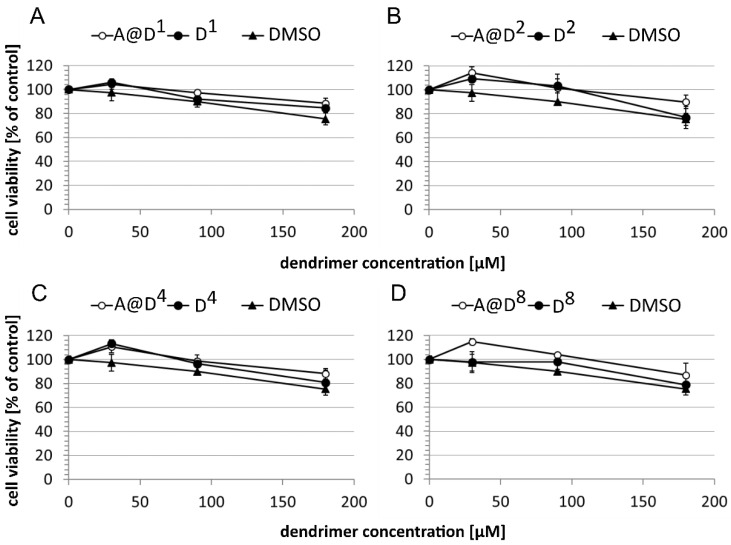
Viability of Caco-2 cells after treatment with G3 PAMAM dendrimer conjugates: (**A**) A@D^1^, D^1^; (**B**) A@D^2^, D^2^; (**C**) A@D^4^, D^4^, and (**D**) A@D^8^, D^8^ at concentrations of 30, 90, and 180 µM. The concentrations of DMSO were 0.5%, 1.5%, and 3%, respectively. Data are presented as means ± SD normalized to untreated control.

**Figure 5:**
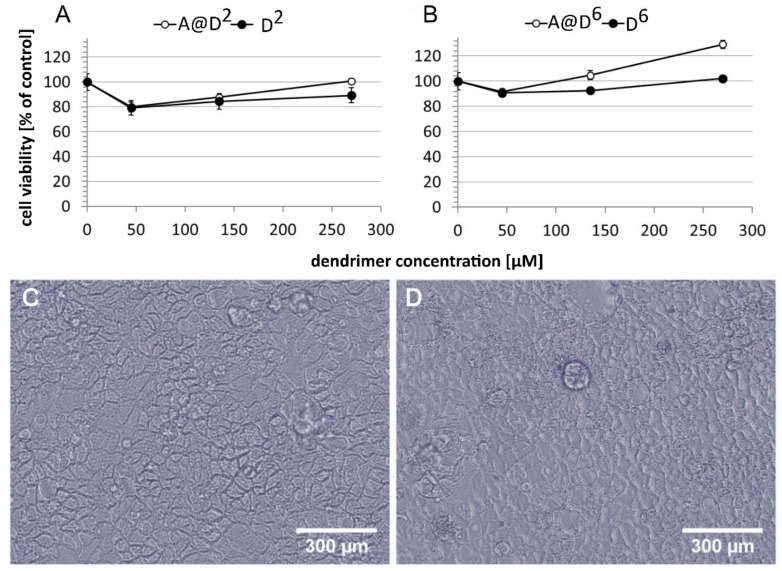
Viability of Caco-2 cells after treatment with aqueous solutions of encapsulates and dendrimer conjugates: (**A**) A@D^2^, D^2^ and (**B**) A@D^6^, D^6^ at concentrations of 45, 135, and 270 µM. Data are presented as means ± SD normalized to untreated control. Images of Caco-2 cells: (**C**) Control and (**D**) incubated with D^2^ at concentration of 270 µM.

To assess the dark cytotoxicity of aqueous and DMSO solutions of PAMAM-biotin conjugates and its ALA@G3^gh^ encapsulates, the MTS cell viability assay was performed. The concentration of dendrimers was normalized to the concentration of ALA in acid-containing conjugates which was equal 180, 540 µM, and 1.08 mM, respectively, in solutions in H_2_O/DMSO (A@D^1^, A@D^2^, A@D^4^, and A@D^8^) and water (A@D^2^ and A@D^6^). The viability of Caco-2 cells treated with dendrimer conjugates at concentrations of 30, 90, and 180 µM in H_2_O/DMSO is shown in Figure 4. Results of the experiments conducted with the use of aqueous solutions of dendrimers at concentrations of 45, 135, and 270 µM were shown in Figure 5. All the conjugates exhibited acceptable cytotoxicity to Caco-2 colorectal adenocarcinoma cells and did not affect the morphology of cells (Figure 5C,D). Dendrimer conjugates containing ALA were not more cytotoxic, indicating low dark cytotoxicity.

#### 2.2.2. Accumulation of Protoporphyrin IX in Caco-2 Cells

**Figure 6:**
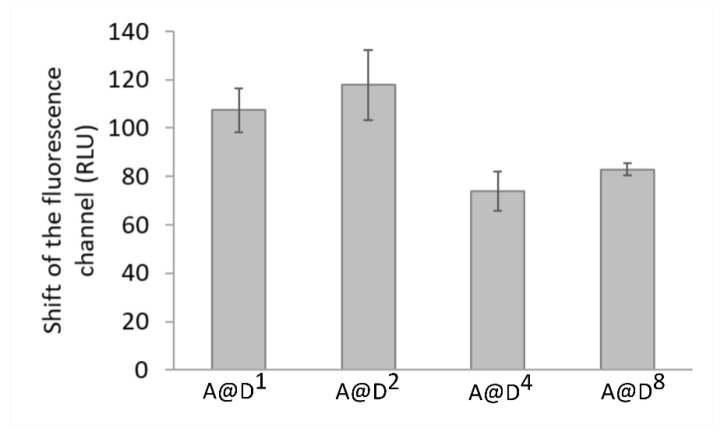
Shift of the mean fluorescence channel of A@D^1^, A@D^2^, A@D^4^, and A@D^8^ encapsulates in relation to conjugates without ALA (D^1^, D^2^, D^4^, and D^8^, respectively). Fluorescence detection wavelength was 605 nm. Data are presented as means ± SD.

To assess the accumulation of Protoporphyrin IX, Caco-2 cells were treated with 180 µM of dendrimers in H_2_O/DMSO (prepared by dilution of stock solutions in DMSO; 6 D^n^ and 36 mM ALA, where n = 1B, 2B, 4B, or 8B). After 24-h incubation, fluorescence at 605 nm was assayed using cytofluorimeter. This wavelength corresponded to the peak of PpIX fluorescence [32]. As expected, the highest fluorescence intensity was observed in cells treated with conjugates containing Protoporphyrin IX precursor–ALA (encapsulates A@D^1^, A@D^2^, A@D^4^, and A@D^8^). The greatest shift of the mean fluorescence channel in relation to host dendrimers was observed for A@D^1^ and A@D^2^ encapsulates (Figure 6), which indicates the highest accumulation of Protoporphyrin IX in cells.

**Figure 7:**
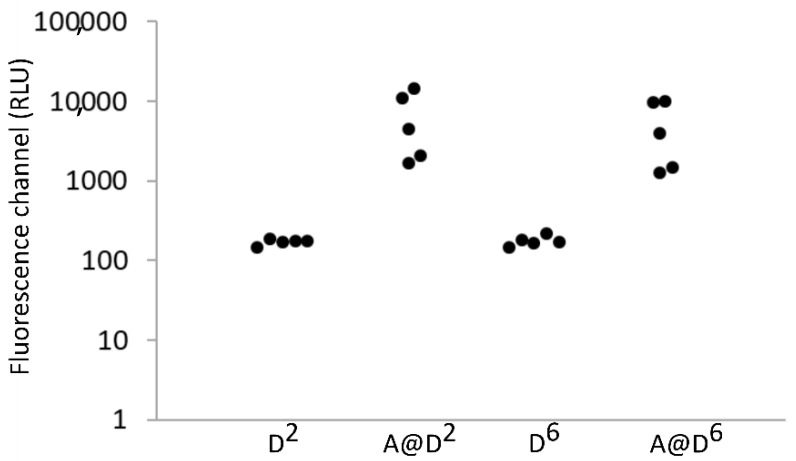
Mean fluorescence channel of D^2^, A@D^2^, D^6^, and A@D^6^ dendrimers upon excitation with 405 nm laser. Fluorescence detection wavelength was 711 nm.

In another experiment, cells were treated with 180 µM of encapsulates in H_2_O/DMSO or with 270 µM of encapsulates in water (Figure 7) (the concentration of ALA was 1.08 mM in case of both solvents). After 24-h incubation, a fluorescence at 711 nm was assayed using flow cytofluorimeter. The 710 nm wavelength corresponded to another peak of PpIX fluorescence [33].

Fluorescence detection at this wavelength provided the greatest values of the mean fluorescence channel of PpIX synthesised from ALA contained in aqueous solutions of dendrimers. Using this method of fluorescence detection, the shift of the mean fluorescence channel for conjugates dissolved in H_2_O/DMSO was about 10 times smaller than in the case of detection at 605 nm. For aqueous solutions of encapsulates, the significant increase of mean fluorescence channel was observed for both A@D^2^ and A@D^6^ (Figure 7). This indicates that 24-h incubation of cells with ALA-containing conjugates is a sufficient time for Protoporphyrin IX synthesis. Short incubations resulted in an undetectable increase of mean fluorescence channel.

Interestingly, we found that an increased number of biotin residues in the host conjugate did not improve the intracellular level of PpIX in comparable conditions. In fact, in the case of host G3^4B24gh^ and G3^8B17gh^, a decrease of the PpIX level was noticed. This inverse effect was probably due to an association of a higher biotin-substituted conjugate. Indeed, we observed considerably higher values of volume-average molecular size of G3^4B24gh^ and G3^8B17gh^ (>6.5 nm in both cases) in comparison with the number-average diameter (5.0 nm both). Considerably, a larger volume-average in relation to the number-average molecular size evidenced the association of dendrimers and was previously found for cytisine-G3^gh^ conjugates [31]. Another reason for such an effect can be related to the elevated efflux of high biotin-substituted conjugates, especially in the presence of DMSO. 

#### 2.2.3. Intracellular Reactive Oxygen Species Level

In order to study the phototoxicity of ALA encapsulates, we examined the level of reactive oxygen species (ROS) induced upon the illumination of treated cells. Caco-2 cells were incubated with conjugates for 24 h and then illuminated with a 655-nm laser beam, corresponding to the PDT window of Protoporphyrin IX. Illumination was performed for (A) 30s (E = 137 mW/cm^2^, H = 4.1 J/cm^2^) and (B) 60s (E = 137 mW/cm^2^, H = 8.2 J/cm^2^). With the applied illumination parameters and ALA concentration, we did not observe the phototoxic effect of conjugates dissolved in DMSO and water upon 1-min illumination. The phototoxic effect, manifested by the ROS production in cells illuminated for 30 s, was observed for A@D^2^ and A@D^6^ conjugates in aqueous solution, not in the presence of traces of DMSO (Table 1).

## 3. Materials and Methods 

### 3.1. Reagents and Methods

All the chemicals used in synthesis of the PAMAM G3 dendrimer and its conjugates as well as 5-aminolevulinic acid hydrochloride were purchased from Merck (KGaA, Darmstadt, Germany).

The 1-D ^1^H and ^13^C NMR spectra as well as 2-D ^1^H-^1^H correlation spectroscopy (COSY), ^1^H-^13^C heteronuclear single quantum correlation (HSQC), and the heteronuclear multiple bond correlation spectra (HMBC), were recorded in deuterated water using Bruker 300 MHz (Rheinstetten, Germany) and worked up with TopSpin 3,5 software at the College of Natural Sciences, University of Rzeszów.

### 3.2. Chemical Syntheses

#### 3.2.1. PAMAM G3 Substituted with Biotin

PAMAM G3 dendrimer was obtained at the 5 millimolar scale according to the procedure published by Tomalia et al. [34], and stored as 20.1 mM solution in methanol for further use. Then G3 was substituted with 1, 2, 4, and 8 equivalents of biotin by stepwise addition of solid *N*-hydroxysuccinimide ester of biotin (NHS-B) into 0.414 g of G3 (60.3 µM) dissolved in 3 mL dimethylsulfoxide (DMSO) with vigorous stirring. The mixture was left at an ambient temperature for 12 h, transferred into dialytic tube (nitrocellulose, MW_cutoff_—3.5 kD), and dialyzed against water for 3 days (5 times 3 dm^3^). Water was evaporated under reduced pressure and products were identified by ^1^H NMR spectroscopy as G3 substituted with average 1, 2, 4, 6, and 8 equivalents of biotin per one PAMAM G3 molecule: G3^1B^, G3^2B^, G3^4B^, G3^6B^, and G3^8B^, respectively. The isolated yield was above 80% in every case.

#### 3.2.2. PAMAM G3 Substituted with Biotin and Glucoheptoamide

The biotin-substituted G3 PAMAM dendrimers were further converted by blocking amine groups in reaction of *ca* 20 µM of G3^1B^, G3^2B^, G3^4B^, G3^6B^, and G3^8B^ with 20% excess of α-*D*-glucoheptono-1,4-lactone (GHL) in relation to terminal amine group of G3. In a typical procedure to the 120 mg of G3^2B^ (16.3 µM), 2 mL DMSO solid GHL was added stepwise (135 mg, 648 µM) with magnetic stirring until it dissolved. The mixture was kept at 50 °C for 6 h and then dialyzed against water for 2 days. Afterwards, water was removed under reduced pressure and solid products were isolated at >80% yield.

The stoichiometry of obtained conjugates was examined by the ^1^H NMR spectroscopy (Figure 2) and it was noticed that not all amine groups were converted into glucoheptoamide derivative. The following conjugates were obtained: G3^1B31gh^, G3^2B27gh^, G3^4B24gh^, G3^6B20gh^, and G3^8B17gh^. Thus, in these conjugates, average 0, 3, 4, 5, and 7 amine groups were left unsubstituted, respectively. We also prepared biotin-free conjugate G3^32gh^, with all primary amine groups converted into glucoheptoamide as described elsewhere [31,35].

The molecular size of conjugates was determined by the DLS method as before [31]. The number-average diameters of G3^32gh^, G3^2B27gh^, and G3^6B21gh^ at pH 5 (0.05 M acetate buffer) were 4.0 (±0.2), 4.2 (±0.3), and 4.4 (±0.3) nm, while the volume-average diameters were 4.6 (±0.2), 5.0 (±0.3), and 5.0 (±0.3) nm, respectively. The size of all conjugates in water were within 1.8–2.0 (±0.3) nm (number-average).

#### 3.2.3. Encapsulation of ALA in the G3^Bgh^ Conjugates

Interaction between the G3^32gh^ conjugate and ALA in aqueous solution was monitored by the ^1^H NMR spectroscopy. Thus, solid ALA was added into an NMR tube containing 5.1 mM G3^32gh^, and NMR spectra were recorded after ALA was dissolved. Addition of ALA was continued until final portion of ALA remained undissolved. The spectrum of the final solution in equilibrium with the precipitate was taken after one day equilibration. The precipitate was separated from the mixture and identified as pure ALA. The final concentration of ALA in the presence of 5.1 G3^32gh^ was 76 mM, which was *ca* twice higher in comparison to the concentration of ALA in a saturated aqueous solution (D_2_O). The ^1^H NMR spectra of starting compounds and mixtures of G3^32gh^ and ALA are presented in Figure 3.

Similar experiments were performed in the case of all G3^1B31gh^, G3^2B27gh^, G3^4B24gh^, and G3^8B17gh^ conjugates and ALA. The results are illustrated by series of ^1^H NMR spectra of G3^4B24gh^ and ALA in D_2_O in Figure 4. In the case of this experiment, the concentration of G3^4B24gh^ conjugate was 6.2 mM, while the final concentration of ALA was *ca* 50 mM in equilibrium with the solid. The solid was isolated and identified as a G3^4B24gh^: ALA 1:8 complex. Similar results were also obtained for other conjugates containing a variable amount of biotin.

Finally, the 6-mM solutions of G3^Bgh^ conjugates and 36 mM ALA in DMSO were prepared and used as stock solution for biological tests. The ^1^H NMR spectra of all solutions were examined after one month storage at room temperature and showed no traces of converted ALA.

In order to determine the stability of ALA@G3^4B24gh^ and other encapsulates, the 5-mM aqueous solution of 8ALA@G3^4B24gh^ encapsulate (10 mL) was dialyzed in a cellulose bag (MW_cutoff_ = 3.5 kDa) against 0.1 M phosphate buffer pH 7.2 (3 dm^3^) four times for 4 h and at every step. The ^1^H NMR spectrum of remaining encapsulate was recorded. The final composition of dialyzed encapsulate was determined as 6ALA@G3^4B24gh^.

The hydrodynamic diameters determined by DLS in aqueous solutions containing G3^32gh^, G3^2B27gh^, and G3^6B21gh^, and 6 equivalents of ALA were 5.0, 5.3, and 5.4 (±0.3) nm, which increased slightly upon addition of further 10 equivalents of ALA into: 5.2, 5.4, and 5.5 (±0.3) nm, respectively. 

### 3.3. Cell Culture Conditions and Materials

Colorectal adenocarcinoma cells (Caco-2) were cultured in eagle medium (Ludwik Hirszfeld Institute of Immunology and Experimental Therapy) supplemented with 10% fetal bovine serum (FBS, Gibco, Thermo Fisher Scientific, Waltham, MA, USA) and 2 mM L-glutamine (Sigma–Aldrich, Saint Louis, MO, USA). Cells were cultured on Petri dishes (Sarstedt AG and Co. KG, Nümbrecht, Germany), flasks, and 24- or 96-well plates (Corning Life Sciences, Kennebunk, ME, USA) at 37 °C in a humidified atmosphere of 5% CO_2_.

### 3.4. Toxicity Studies

Caco-2 cells were seeded on a 96-well plate (Corning Life Sciences, Kennebunk, ME, USA). Cells were treated with ALA encapsulates at concentrations of 30, 90, and 180 µM in H_2_O/DMSO and with aqueous solutions of dendrimers at concentrations of 45, 135, and 270 µM (Table 2). After 24-h incubation at 37 °C, the viability of cells was evaluated with an MTS assay, and 15 µL of CellTiter 96® AQueous One Solution Reagent (Promega, Madison, WI, USA) was added to each well. After 2 h at 37 °C in a humidified, 5% CO_2_ atmosphere, the absorbance at 490 nm was recorded using the Wallac 1420 Victor 2 plate reader (Perkin Elmer, Waltham, MA, USA). Cells incubated with H_2_O/DMSO (Sigma–Aldrich, Saint Louis, MO, USA) at the same dilutions as the dendrimer served as a solvent control.

### 3.5. Accumulation of Protoporphyrin IX in Caco-2 Cells

Caco-2 cells were seeded on a 24-well plate (Corning Life Sciences, Kennebunk, ME, USA). Cells were treated with A@D^n^ conjugates at a concentration of 180 µM (solutions in H_2_O/DMSO) and at a concentration of 270 µM (aqueous solutions). After 24-h incubation at 37 °C in a humidified, 5% CO_2_ atmosphere, cells were harvested and washed with PBS (phosphate-buffered saline solution, Ludwik Hirszfeld Institute of Immunology and Experimental Therapy). Cells were analyzed using LSRFortessa flow cytometer (Becton Dickinson, Franklin Lakes, NJ, USA). Excitation and emission wavelengths were chosen to detect the presence of Protoporphyrin IX in cells: a 405-nm laser was used as a fluorescence excitation source, and the fluorescence was measured at 605 and 710 nm. For data analysis, Flowing Software 2.5.1 was used (Turku University, Turku, Finland).

### 3.6. Intracellular Reactive Oxygen Species Level

Reactive oxygen species (ROS) induction was assessed by measuring the 488-/530-nm fluorescence of H2DCFDA (6-carboxy-20, 70-dichlorodihydrofluorescein diacetate, di(acetoxymethyl ester), Molecular Probes, Thermo Fisher Scientific, Inc., Waltham, MA, USA. In this regard, Caco-2 cells were seeded on a 24-well plate (Corning Life Sciences, Kennebunk, ME, USA). Cells were treated with A@D^n^ encapsulates at concentration of 180 µM (solutions in H_2_O/DMSO) and at a concentration of 270 µM (aqueous solutions). After 24-h incubation at 37 °C in a humidified, 5% CO_2_ atmosphere, culture medium was harvested. Cells were stained with 10 µM of H2DCFDA for 30 min in PBS with 10% FBS and washed twice with warm PBS with 2.5% FBS. Next, cells were illuminated ((A) λ = 655 nm, E = 137 mW/cm^2^, H = 4.1 J/cm^2^ and (B) λ = 655 nm, E = 137 mW/cm^2^, H = 8.2 J/cm^2^). Then cells were harvested, washed in PBS, and analyzed using LSRFortessa flow cytometer (Becton Dickinson, Franklin Lakes, NJ, US). For data analysis, Flowing Software 2.5.1 was used (Turku University, Turku, Finland).

## 4. Conclusions

Nanosized dendrimers are currently tested as drug delivery systems in many laboratories. The current state of nanotechnology applications in colorectal cancer has been recently reviewed, including clinical trials and status [11]. The chitosan nanoparticles loaded with ALA were already demonstrated as a photosensitizer delivery system (PSDS) and convenient tool for fluorescent endoscopic detection of colorectal cancer cells [12]. The chitosan was further equipped with folic acid for targeting cell membrane and enhancing nanoparticle endocytosis via the folate receptor [36]. Generally, a dendrimer-based PSDS can be constructed either as encapsulates of PS in dendrimer [37,38,39,40] or conjugates of the dendrimer with the PS molecule attached covalently [31]. In both strategies, the dendrimeric carrier can be designed to enter the cell; in the latter, the active molecule of PS needs to be cleaved from the conjugate [41]. Another important factor of PSDS is to equip the carrier with a cancer cell membrane-targeting molecule, which is commonly folate [37] or biotin [29]. We used the glucoheptoamidated PAMAM G3, equipped with variable equivalents of biotin, to address the encapsulated ALA to colorectal adenocarcinoma cells in vitro using the Caco-2 line. From previous studies, we know that the G3^Bgh^ carriers enter the cells of normal fibroblasts (BJ), squamous cell carcinoma (SCC-15), and glioblastoma (U-118 MG) within 24 h in a time- and concentration-dependent manner and are 3–4 times less cytotoxic than the non-biotinylated carrier. All cell lines survived a 50-µM concentration of G3^Bgh^ as well as keratinocytes (HaCat) [29,42]. 

In this paper, we found that the third generation polyamidoamine dendrimer, with amide-linked biotin and glucoheptoamide substituents (G3^Bgh^), is a highly effective host to encapsulate 12-6 molecules of 5-aminolevulinic acid (ALA) in water. Aminolevulinic acid was deprotonated and hydrogen cation was transferred from its carboxylic group into internal ternary nitrogen atoms of the host. The encapsulated guest molecules were bound by ionic interaction and were released slowly in neutral pH. The payload of encapsulates depended on the number of biotin residues in the conjugate and equaled 12 for one biotin-containing conjugate. 

The conjugates containing 2 to 6 biotin residues were able to encapsulate at least 7 guest molecules of ALA. When aqueous solutions were applied to colorectal adenocarcinoma cells (Caco-2 line) at a 1-mM concentration of ALA for 24 h, the rapid increase of Protoporphyrin IX was observed. The Protoporphyrin IX-induced cells produced single oxygen upon 30-s irradiation with a 655-nm laser pulse. Both phenomena accompanied the regular response and photocytotoxicity pattern for photodynamic anticancer therapy, including colorectal cancer [11,12].

The encapsulates of ALA in G3^Bgh^ offered the possibility to use this PSDS in local treatment if the concentration of deposited encapsulates was higher. In order to estimate the effectiveness of the designed PSDS, the PK profiles were needed from in vivo studies on model animals. In local PS delivery, the concentration of G3^Bgh^ could be higher than 0.3 mM; thus, the ALA concentration could be at least 3 mM. Another issue to optimize in vivo is the number of biotins in G3^Bgh^; here we did not find the difference in Protoporphyrin IX level between the 2 and 6 biotin-containing host. The elaborated PSDS is currently being tested for treatment of the skin impairments encountered in the introduction [43].

## Figures and Tables

**Table 1:** Shift of the mean fluorescence channel of A@D^1^, A@D^2^, A@D^4^, A@D^8^ (aqueous/DMSO solution), and A@D^2^ and A@D^6^ (in aqueous solution) dendrimers in relation to conjugates without ALA (D^1^, D^2^, D^4^, D^8^, and D^2^ and D^6^, respectively). The parameters of cell illumination were: (A) λ = 655 nm, E = 137 mW/cm^2^, H = 4.1 J/cm^2^, and (B) λ = 655 nm, E = 137 mW/cm^2^, H = 8.2 J/cm^2^. Excitation wavelength was 488 nm; fluorescence was detected at 530 nm.

	Shift of the Fluorescence Channel of H2DCFDA (RLU)					
	H_2_O/DMSO Solution		in H_2_O (DMSO Excluded)			
Experiment	A@D^2^	A@D^6^	A@D^1^	A@D^2^	A@D^4^	A@D^8^
A (30 s illumination)	263.28	443.29				
B (60 s illumination)	−206.1	44.49	−529.43	−363.42	−514.6	−1084.1

**Table 2:** Concentrations of dendrimers, ALA, and DMSO in samples used in toxicity studies.

Compound	Dendrimer Concentration	ALA Concentration	DMSO Concentration
D^1^, D^2^, D^4^, D^8^	30 µM	0	0.5%
	90 µM	0	1.5%
	180 µM	0	3%
A@D^1^, A@D^2^, A@D^4^, A@D^8^	30 µM	180 µM	0.5%
	90 µM	540 µM	1.5%
	180 µM	1.08 mM	3%
D^2^, D^6^	45 µM	0	–
	135 µM	0	
	270 µM	0	
A@D^2^, A@D^6^	45 µM	180 µM	
	135 µM	540 µM	
	270 µM	1.08 mM	

## Data Availability

The data presented in this study are available on request from the corresponding author. The data are not publicly available due to data are collected and stored in 4 different laboratories, encountered in affiliations of all authors. The corresponding author is responsible for transferring the data.
